# Sharing perspectives and experiences of doctoral fellows in the first cohort of Consortium for Advanced Research Training in Africa: 2011–2014

**DOI:** 10.3402/gha.v7.25127

**Published:** 2014-09-29

**Authors:** Babatunde Adedokun, Peter Nyasulu, Fresier Maseko, Sunday Adedini, Joshua Akinyemi, Sulaimon Afolabi, Nicole de Wet, Adedokun Sulaimon, Caroline Sambai, Wells Utembe, Rose Opiyo, Taofeek Awotidebe, Esnat Chirwa, Esther Nabakwe, François Niragire, Dieudonné Uwizeye, Celine Niwemahoro, Mphatso Kamndaya, Victoria Mwakalinga, Kennedy Otwombe

**Affiliations:** 1 Department of Epidemiology and Medical Statistics, Faculty of Public Health, College of Medicine, University of Ibadan, Ibadan, Nigeria; 2 School of Public Health, Faculty of Health Sciences, University of the Witwatersrand, Johannesburg, South Africa; 3 School of Health Sciences, Monash University, Johannesburg, South Africa; 4 College of Medicine, Faculty of Community Health, University of Malawi, Lilongwe, Malawi; 5 Demography and Social Statistics, Obafemi Awolowo University Ile-Ife, Nigeria; 6 Demography and Population Studies, Faculty of Humanities, University of the Witwatersrand, Johannesburg, South Africa; 7 MRC/Wits Rural Public Health and Health Transitions Research Unit (Agincourt), School of Public Health, Faculty of Health Sciences, University of the Witwatersrand, Johannesburg, South Africa; 8 Department of Literature, Theatre and Film Studies, Moi University, Eldoret, Kenya; 9 Department of Physics and Biochemical Sciences, Malawi Polytechnic, University of Malawi, Blantyre, Malawi; 10 School of Public Health, University of Nairobi, Nairobi, Kenya; 11 Department of Medical Rehabilitation, College of Health Sciences, Obafemi Awolowo University, Ile-Ife, Nigeria; 12 Department of Human Kinetics and Health Education, Faculty of Education, University of Ibadan, Ibadan, Nigeria; 13 Department of Mathematics and Statistics, University of Malawi, Blantyre, Malawi; 14 MRC/Wits Developmental Pathways for Health Research Unit, School of Clinical Medicine, Faculty of Health Sciences, University of the Witwatersrand, Johannesburg, South Africa; 15 Department of Child Health and Pediatrics, College of Health Sciences, Moi University, Eldoret, Kenya; 16 Department of Anthropology and Human Ecology, School of Arts and Social Sciences, Moi University, Eldoret, Kenya; 17 Department of Applied Statistics, University of Rwanda, Huye, Rwanda; 18 Department of Sustainable Development, University of Rwanda, Huye, Rwanda; 19 Centre for Population Studies and Research, University of Dar es Salaam, Dar es Salaam, Tanzania; 20 Ifakara Health Institute, Dar es Salaam, Tanzania; 21 Perinatal HIV Research Unit, Faculty of Health Sciences, University of the Witwatersrand, Johannesburg, South Africa

**Keywords:** CARTA, JAS, doctoral fellow, first cohort, advanced research training

## Abstract

**Background:**

Resolution of public health problems in Africa remains a challenge because of insufficient skilled human resource capacity. The Consortium for Advanced Research Training in Africa (CARTA) was established to enhance capacity in multi-disciplinary health research that will make a positive impact on population health in Africa.

**Objective:**

The first cohort of the CARTA program describes their perspectives and experiences during the 4 years of fellowship and puts forward suggestions for future progress and direction of research in Africa.

**Conclusions:**

The model of training as shown by the CARTA program is an effective model of research capacity building in African academic institutions. An expansion of the program is therefore warranted to reach out to more African academics in search of advanced research training.

The Consortium for Advanced Research and Training in Africa (CARTA) was founded in 2010 as a partnership between African institutions and some northern partners with the overarching goal of building research capacity in Africa and encouraging multi-country and multi-disciplinary research that could bring a positive impact on population health within the continent (1). CARTA’s primary objective is to strengthen research infrastructure and build capacity in African universities through enhancement of doctoral training programs (1). The idea behind CARTA is unique in a number of ways; it is arguably the first partnership initiated by organizations based in Africa and primarily involved in research on the continent. Second, the model of bringing together young scientists across sub-Saharan Africa is unprecedented in the region. Although there have been several consortia between African institutions and northern partners (2–5), none of these have had the distinction of mentoring doctoral students from various disciplines. The continent of Africa faces enormous public health challenges. Hence, advanced research training as propagated by the CARTA program is essential in identifying effective interventions that will improve the livelihood of communities in Africa.

The purpose of this paper is therefore to share perspectives and learning experiences of the first cohort of doctoral fellows that participated in the CARTA program over a period of 4 years and highlight the future progress and direction of research training in Africa.

## Learning platform for professional development

The CARTA program provided a platform for research training and professional development among the doctoral fellows through involvement in the Joint Advanced Seminars (JAS). The entire program consisted of four JAS sessions that were conducted between March 2011 and March 2014 (1).

**Fig. 1:**
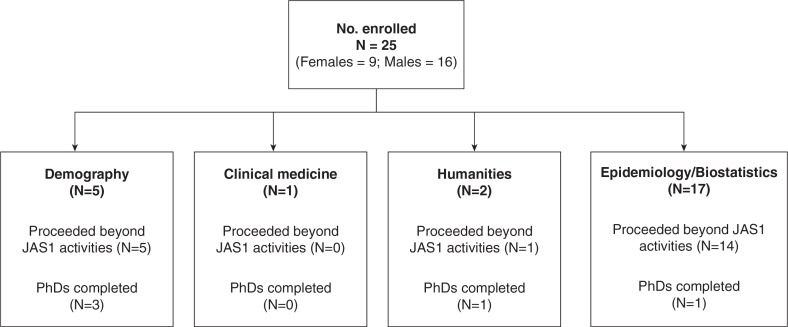
Disposition flow chart for various disciplines of doctoral fellows, cohort 1.

The first cohort of doctoral fellows included 25 researchers from various disciplines, that is, epidemiology, biostatistics, demography, sociology, health management, health promotion, health systems, environmental sciences, and urban health (Fig. 1). The doctoral fellows were from six African countries: Kenya, Malawi, Nigeria, Rwanda, South Africa, Tanzania, and Uganda. Five fellows did not continue beyond the second JAS, and the remaining 20 fellows were still with CARTA by the end of the fourth JAS.

## Learning equipment and skills development

There were several benefits derived from the CARTA program, the significant ones being those related to skills building. At the commencement of the JAS, essential equipment were provided to each doctoral fellow which included laptop computers, and the software programs ‘NVIVO, STATA, and endnote’, to facilitate learning and skills development. Such provision created a conducive environment for fellows to advance their skills in quantitative and qualitative analysis of research data as well as effective referencing of published literature. To ensure proficiency in utilization of the learning equipment, training sessions were provided by senior researchers. In the process, individual fellows with a quantitative background gained skills in qualitative data analysis and vice-versa. This model of learning provided an opportunity for fellows to be able to effectively engage in research using either of these methodologies.

## Research dissemination

The CARTA program provided research support such as travel grants to enable fellows to disseminate their research findings in both local and international conferences (Table 1). Within the 4-year period, 64 publications were authored or co-authored by CARTA fellows. The CARTA program also provided funding for implementation of the PhD research projects as well as a network platform for collaboration with experts from partner institutions who were co-supervising doctoral theses. In addition, the program provided fellows with broader access to journal articles and other scholarly resources which fellows would otherwise not have accessed.

**Table 1:** Local and international conferences attended by doctoral fellows, cohort 1

Conference	City, Country	Year
7th Public Health Association of South Africa conference	Johannesburg, South Africa	2011
6th APC of Union for African Population Studies	Ouagadougou, Burkina Faso	2011
15th International Congress of Infectious Diseases	Bangkok, Thailand	2012
4th Infection Control Africa Network International Conference	Cape Town, South Africa	2012
Population Association of America Annual Meeting	San Francisco, USA	2012
2nd Asia Population Conference	Bangkok, Thailand	2012
Wellcome Trust 6th Meeting of the Directors of the African Institutions Initiative	Accra, Ghana	2012
Infection Prevention Network	Mombasa, Kenya	2013
27th International Union for the Scientific Study of Population	Busan, South Korea	2013
International Symposium on Intra-urban Dynamics and Health	Paris, France	2013
Scientific Symposium for Emerging Scholars in Health	Nairobi, Kenya	2013
4th Annual East Africa Health and Scientific Conference,and International Health Exhibition and Trade Fair	Kigali, Rwanda	2013
9th Public Health Association of South Africa Conference	Cape Town, South Africa	2013
Urbanisation, HIV and Inequality Conference	Cape Town, South Africa	2013
17th International Conference on AIDS and STIs in Africa	Cape Town, South Africa	2013
20th Conference on Retroviruses and Opportunistic Infections	Atlanta, USA	2013
6th MIM Pan-African Malaria Conference	Durban, South Africa	2013
14th Congress of International Federation of Infection Control	St. Juliana, Malta	2014
Population Association of America Annual Meeting	Boston, USA	2014
6th Africa Conference on Sexual Health and Rights	Yaoundé, Cameroon	2014

## Impact of CARTA program on doctoral research progress

At the end of the third year of the program, five fellows had successfully completed their PhD theses and the remaining fellows had made advanced progress and were on course to completion. This is unprecedented progress in the African context especially where doctoral degrees take long to complete (6, 7). The observed progress on time-to-completion might be due to the CARTA program working closely with fellows’ home institutions by ensuring reduction of teaching duties and other departmental activities in the various CARTA-affiliated universities.

## Institutional research collaborations and networking

In addition to the quantitative measures of success highlighted above, there were several other opportunities that were made available that fellows cherished and benefitted from. Fellows had the opportunity to network with senior researchers from highly reputable institutions and created a platform for research collaboration. In addition, there were several informal discussions among fellows that culminated into research collaborations focusing on how to effectively tackle the various public health challenges facing Africa.

Another learning experience shared by fellows was the realization of the post-PhD engagement in research and academic leadership in African institutions. The JAS sessions on leadership skills, teaching roles, policy engagement, and communication, further highlighted the multiple roles that the contemporary African academic and researcher needs to play in influencing government policy (8) thereby acting as an agent of social change.

## Challenges and suggestions for improvement

Despite the major benefits mentioned above, fellows were faced with unprecedented challenges that were an obstacle to academic engagement. These included combining doctoral training and institutional responsibilities which substantially impeded research progress. As previously reported, teaching load in African universities is usually heavy (9). As much as other institutions within the CARTA program network provided fellows time for their doctoral research, other institutions did not comply with this requirement. This might provide a plausible explanation behind the 20% attrition rate observed. Therefore, there is need to reinforce the agreement between CARTA and participating institutions on providing time for research training activities, such as CARTA fellows registering for PhD in institutions other than their home institutions and CARTA making provisions for PhD writing retreats outside of the JAS sessions.

It was observed that fellows pursuing doctoral research outside of their home institutions made substantial progress compared with fellows registered in their home institutions. This calls for a realignment of doctoral supervision in home institutions to allow for enhanced supervision that provides a suitable environment for progress and timely completion of the doctoral degree. The African continent needs more initiatives like the CARTA program that provides advanced research training to junior African academics so that substantial capacity and skills are developed in Africa that could be applied to tackle public health challenges. Success stories have been reported from other north–south partnerships in Africa (2, 10–13) signifying the value of partnerships on the African continent.

In addition, the CARTA model should be expanded to include other countries within Africa. Currently, the CARTA program includes nine universities in six countries. The ultimate goal should be to include most universities from sub-Saharan and North Africa. However, inadequate funding is a major threat to sustainability or expansion of partnerships for capacity building in Africa (14). For this reason, we propose that the universities currently in the CARTA program be more actively involved in raising funds for research training initiatives in their institutions.

## Conclusion

The CARTA program created a platform that provided doctoral fellows with an opportunity for networking and research collaboration in public and population health in Africa. There is no doubt that this program has been successful and has demonstrated substantial enhancement of knowledge and technical skills required for carrying out quality research. In addition, the CARTA platform enabled opportunities for networking among African researchers and engagement with policy makers, the media, and other stakeholders who facilitate translation of research findings to policy (15). A great opportunity exists for African leaders to support and invest in similar programs while at the same time ensuring sustainability (16).
